# Supplementary material for: Differences in the fitness effects of traded resources shape traits and persistence in multi-mutualist communities
Source: PLoS One. 2026 Feb 3;21(2):e0340707. doi: 10.1371/journal.pone.0340707 (PMC12867262; doi:10.1371/journal.pone.0340707)
Supplement: S1 Table — (DOCX) [file pone.0340707.s001.docx]

| **Strain ID** | **Genotype** | **Selective plate** |
| --- | --- | --- |
| RY1069 | MATa ste3ΔkanMX4 lys2Δ0 leu2Δ0 ura3Δ0 ADE4OP | SD-Ade-His-Trp |
| RY1070 | MATa ste3ΔkanMX4 lys2Δ0 leu2Δ0 ura3Δ0 ADE4OP | SD-Ade-His-Trp |
| RY1063 | MATa ste3ΔkanMX4 lys2Δ0 his3Δ1 ura3Δ0 ADE4OP | SD-Ade-Leu-Trp |
| RY1064 | MATa ste3ΔkanMX4 lys2Δ0 his3Δ1 ura3Δ0 ADE4OP | SD-Ade-Leu-Trp |
| RY1057 | MATa ste3ΔkanMX4 lys2Δ0 his3Δ1 leu2Δ0 ADE4OP | SD-Ade-Ura-Trp |
| RY1058 | MATa ste3ΔkanMX4 lys2Δ0 his3Δ1 leu2Δ0 ADE4OP | SD-Ade-Ura-Trp |
| RY1081 | MATa ste3ΔkanMX4 lys2Δ0 leu2Δ0 ura3Δ0 trp1Δ63HygBR ADE4OP | SD-Ade-His+Hyg |
| RY1082 | MATa ste3ΔkanMX4 lys2Δ0 leu2Δ0 ura3Δ0 trp1Δ63HygBR ADE4OP | SD-Ade-His+Hyg |
| RY1045 | MATa ste3ΔkanMX4 ade8Δ0 leu2Δ0 ura3Δ0 LYS21OP | SD-Lys-His |
| RY1046 | MATa ste3ΔkanMX4 ade8Δ0 leu2Δ0 ura3Δ0 LYS21OP | SD-Lys-His |
| RY1083 | MATa ste3ΔkanMX4 ade8Δ0 his3Δ1 ura3Δ0 trp1Δ63HygBR LYS21OP | SD-Lys-Leu+Hyg |
| RY1084 | MATa ste3ΔkanMX4 ade8Δ0 his3Δ1 ura3Δ0 trp1Δ63HygBR LYS21OP | SD-Lys-Leu+Hyg |
| RY1085 | MATa ste3ΔkanMX4 ade8Δ0 his3Δ1 leu2Δ0 trp1Δ63HygBR LYS21OP | SD-Lys-Ura+Hyg |
| RY1086 | MATa ste3ΔkanMX4 ade8Δ0 his3Δ1 leu2Δ0 trp1Δ63HygBR LYS21OP | SD-Lys-Ura+Hyg |
| RY1039 | MATa ste3ΔkanMX4 ade8Δ0 his3Δ1 leu2Δ0 LYS21OP | SD-Lys-Ura-Trp |
| RY1040 | MATa ste3ΔkanMX4 ade8Δ0 his3Δ1 leu2Δ0 LYS21OP | SD-Lys-Ura-Trp |

Table S1: Yeast strains used in this study.
