# Supplementary material for: Differences in the fitness effects of traded resources shape traits and persistence in multi-mutualist communities
Source: PLoS One. 2026 Feb 3;21(2):e0340707. doi: 10.1371/journal.pone.0340707 (PMC12867262; doi:10.1371/journal.pone.0340707)
Supplement: S2 Table — “X” indicates the absence of a strain. In no external resource (obligate) media, only three communities retained all eight strains by the end of the four-week period. Therefore, we also considered communities with fewer than eight strains. (DOCX) [file pone.0340707.s002.docx]

| **Resources** | **Community composition** |
| --- | --- |
| External resources present | \| AdeOP:1057,1063,1069,1082 \| \| --- \| \| LysOP:1039,1045,1084,1085 \| |
|  | \| AdeOP:1058,1063,1069,1081 \| \| --- \| \| LysOP: 1039,1046,1084,1085 \| |
|  | \| AdeOP:1057,1064,1069,1082 \| \| --- \| \| LysOP: 1039,1046,1084,1086 \| |
|  | \| AdeOP:1058,1064,1069,1082 \| \| --- \| \| LysOP: 1040,1046,1083,1085 \| |
|  | \| AdeOP:1057,1063,1069,1082 \| \| --- \| \| LysOP: 1039,1045,1083,1085 \| |
|  | \| AdeOP:1057,1063,1069,1082 \| \| --- \| \| LysOP: 1040,1046,1083,1086 \| |
|  | \| AdeOP:1058,1063,1069,1081 \| \| --- \| \| LysOP: 1039,1045,1083,1085 \| |
|  | \| AdeOP:1057,1064,1070,1081 \| \| --- \| \| LysOP: 1040,1046,1083,1086 \| |
| No external resource present | \| AdeOP:1057,1063,1070, X \| \| --- \| \| LysOP: 1040,1045,1084,1085 \| |
|  | \| AdeOP: X, 1064, X,1081 \| \| --- \| \| LysOP: 1039,1045,1083,1085 \| |
|  | \| AdeOP:1058,1063,1069,1081 \| \| --- \| \| LysOP: 1040,1046,1083,1086 \| |
|  | \| AdeOP: X, 1064,1070,1082 \| \| --- \| \| LysOP: 1040,1046,1083,1086 \| |
|  | \| AdeOP:1057,1063,1070,1082 \| \| --- \| \| LysOP: X, 1046,1083,1086 \| |
|  | \| AdeOP:1057,1063,1069,1082 \| \| --- \| \| LysOP: 1039,1045,1083,1086 \| |
|  | \| AdeOP:1057,1063,1069,1082 \| \| --- \| \| LysOP: 1039,1045,1084,1085 \| |
|  | \| AdeOP: X, 1063, X, 1070 \| \| --- \| \| LysOP: 1040, X, 1083,1085 \| |

Table S2: Community of yeast mutualists used for phenotyping. “X” indicates the absence of a strain. In no external resource (obligate) media, only three communities retained all eight strains by the end of the four-week period. Therefore, we also considered communities with fewer than eight strains.
