# Supplementary figures and images for: Differences in the fitness effects of traded resources shape traits and persistence in multi-mutualist communities
Source: PLoS One. 2026 Feb 3;21(2):e0340707. doi: 10.1371/journal.pone.0340707 (PMC12867262; doi:10.1371/journal.pone.0340707)

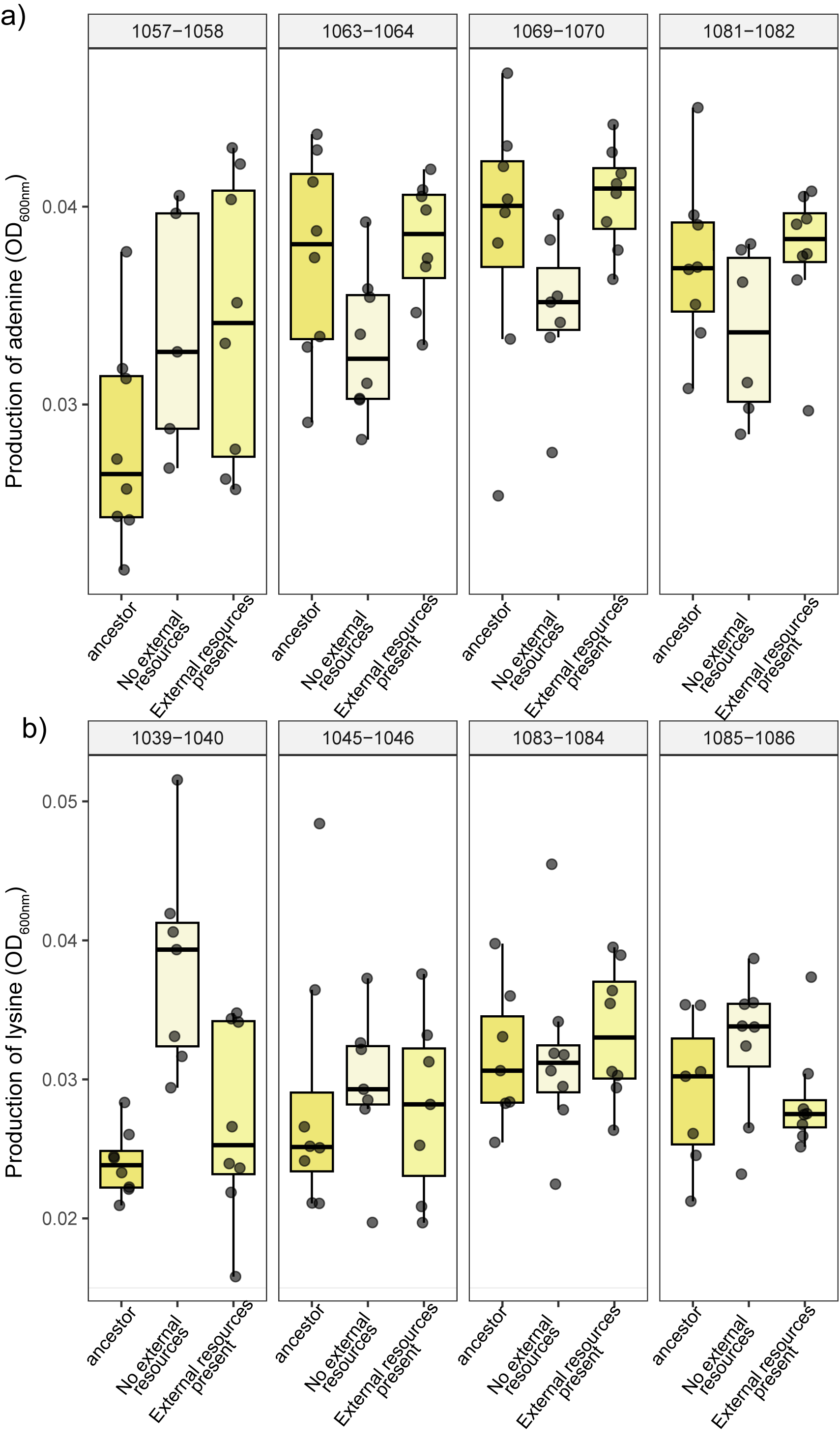

Supplement: S1 Fig — (TIF) [file pone.0340707.s003.tif]
