# Supplementary material for: Differences in the fitness effects of traded resources shape traits and persistence in multi-mutualist communities
Source: PLoS One. 2026 Feb 3;21(2):e0340707. doi: 10.1371/journal.pone.0340707 (PMC12867262; doi:10.1371/journal.pone.0340707)
Supplement: Attachment — Submitted filename: Review_PONE-D-25-49040.docx [file pone.0340707.s004.docx]

The article investigates how fitness asymmetries in exchanged resources shape the evolutionary dynamics and persistence of species using synthetic multi-mutualist yeast communities. The authors have used 8 genetically distinct strains of *Saccharomyces cerevisiae* that either overproduce adenine or lysine but cannot produce the other, the authors simulate mutualism under environments with and without external nutrient supplementation. The study integrates the ecological persistence with trait evolution under different resource contexts, providing direct experimental evidence for how asymmetric fitness consequences can shape mutualism stability. The study is well executed and methodologically rigorous.

**Below are my specific comments to improve clarity and presentation. With these minor changes the manuscript is acceptable for publication.**

1) I would recommend the authors to better represent the model being studied. The current figure is confusing and hard to comprehend. My suggestion is to have a better schematic or flow diagram in the Methods section illustrating how the strains were mixed together, why 96 distinct communities were developed, and why this resulted in 288 evolving cultures under various conditions. This would make the design much more understandable to readers.Maintain clear color codes for genetically different strains.
2) Further, instead of a section named as “strains used in this study” it could be more like “ synthetic model of multi-mutualist communities” . This would emphasize that the work is not only about the strains themselves but about the synthetic mutualism model being tested.
3) While the use of a growth-based bioassay to estimate adenine and lysine overproduction is valid and functionally relevant, direct quantification (e.g., HPLC, LC-MS, or kit-based metabolite assays) would provide complementary information on exact metabolite levels. I encourage the authors to comment on the choice of indirect quantification based method.
4) In the "Overproduction measurements" part, Authors have stated that the LysOPs were heat-shocked to release lysine, which is released primarily upon cell death. For the AdeOPs, they just harvested the filtrate. A minor, complementary experiment would be to subject the AdeOPs to the same heat shock and filtration procedure to determine whether it produces more or a greater amount of adenine that could then be utilized to ascertain that adenine is actually released more quickly and more easily than lysine. This could be a minor addition to the procedures. In the case that this is not done, please clarify it in the methodology or discussion

**Minor edits:**

- Line no : 317 (stains to strains )
- Ensure consistency in figure references (e.g., “Fig 5a” vs. “Figure 5a”).
- A general proofreading pass to correct minor typographical and grammatical errors will improve readability.
- Standardize terminology (sometimes “mutualistic goods,” sometimes “traded resources”).
