# Supplementary material for: Differences in the fitness effects of traded resources shape traits and persistence in multi-mutualist communities
Source: PLoS One. 2026 Feb 3;21(2):e0340707. doi: 10.1371/journal.pone.0340707 (PMC12867262; doi:10.1371/journal.pone.0340707)
Supplement: Attachment — Submitted filename: Response_to_Reviewers.docx [file pone.0340707.s006.docx]

**Comments to Reviewers**

We sincerely thank the reviewers for their thoughtful and constructive comments, which have helped us strengthen the manuscript. Below, we provide point-by-point responses and describe the corresponding revisions.

**Reviewer #1:**

The article investigates how fitness asymmetries in exchanged resources shape the evolutionary dynamics and persistence of species using synthetic multi-mutualist yeast communities. The authors have used 8 genetically distinct strains of *Saccharomyces cerevisiae* that either overproduce adenine or lysine but cannot produce the other, the authors simulate mutualism under environments with and without external nutrient supplementation. The study integrates the ecological persistence with trait evolution under different resource contexts, providing direct experimental evidence for how asymmetric fitness consequences can shape mutualism stability. The study is well executed and methodologically rigorous.

**Below are my specific comments to improve clarity and presentation. With these minor changes the manuscript is acceptable for publication.**

**Comment 1:** I would recommend the authors to better represent the model being studied. The current figure is confusing and hard to comprehend. My suggestion is to have a better schematic or flow diagram in the Methods section illustrating how the strains were mixed together, why 96 distinct communities were developed, and why this resulted in 288 evolving cultures under various conditions. This would make the design much more understandable to readers. Maintain clear color codes for genetically different strains.

**Response 1:** We understand reviewer’s concern. We have now updated figure 1 for better representation.

**Comment 2:** Further, instead of a section named as “strains used in this study” it could be more like “synthetic model of multi-mutualist communities”. This would emphasize that the work is not only about the strains themselves but about the synthetic mutualism model being tested.

**Response 2:** We have changed the “Strains used in this study” to “Synthetic model of multi-mutualist communities”

**Comment 3:** While the use of a growth-based bioassay to estimate adenine and lysine overproduction is valid and functionally relevant, direct quantification (e.g., HPLC, LC-MS, or kit-based metabolite assays) would provide complementary information on exact metabolite levels. I encourage the authors to comment on the choice of indirect quantification-based method.

**Response 3:** We thank the reviewer for this insightful comment. We agree that direct quantification methods such as HPLC or LC-MS would yield absolute concentrations of adenine and lysine and provide a more direct measure of metabolite production. However, we used a growth-based bioassay to estimate adenine and lysine overproduction because it provides a functional measure of the metabolites available to mutualistic partners. This indirect approach integrates both metabolite release and biological accessibility, offering an ecologically relevant estimate of the exchangeable resource. Our study focuses on the relative functional differences among treatments rather than absolute metabolite levels. Moreover, this assay is well-validated for *Saccharomyces cerevisiae* auxotrophs (e.g., Vidal et al. 2020; Shou et al. 2007), allowing us to maintain consistency with prior studies. Additionally, we explored this possibility in the early stages of working with this system, but realized the number of samples we would have to process would make it monetarily prohibitive.

**Comment 4:** In the "Overproduction measurements" part, Authors have stated that the LysOPs were heat-shocked to release lysine, which is released primarily upon cell death. For the AdeOPs, they just harvested the filtrate. A minor, complementary experiment would be to subject the AdeOPs to the same heat shock and filtration procedure to determine whether it produces more or a greater amount of adenine that could then be utilized to ascertain that adenine is actually released more quickly and more easily than lysine. This could be a minor addition to the procedures. In the case that this is not done, please clarify it in the methodology or discussion

**Response 4:** We thank the reviewer for this suggestion. We agree that symmetric extraction protocols could improve comparability between overproducer types. However, adenine and lysine differ fundamentally in their cellular release mechanisms. Adenine is secreted continuously into the medium by AdeOP strains during active growth, while lysine is largely retained intracellularly and released upon cell death and lysis. For this reason, we directly harvested the AdeOP filtrate without heat shock, which accurately represents the biologically available adenine under growth conditions. We have now clarified this rationale in the Methods section (line 243-246):

“We did not heat shock the adenine overproducers so that we would have a more accurate estimate of the adenine excreted without the addition of adenine from disrupted cells. Thus, separate extraction protocols were used to accurately represent biologically available metabolite levels for each overproducer type.”

**Minor edits:**

**Comment 5:** Line no: 317 (stains to strains )

**Response 5:** Changed “stains” to “strains” (now in line 337).

**Comment 6:** Ensure consistency in figure references (e.g., “Fig 5a” vs. “Figure 5a”).

**Response 6:** We have used “Fig” across entire manuscript.

**Comment 7:** A general proofreading pass to correct minor typographical and grammatical errors will improve readability.

**Response 7:** We thank the reviewer for this suggestion. The entire manuscript has been carefully proofread to correct typographical and grammatical errors, ensuring improved clarity and readability.

**Comment 8:** Standardize terminology (sometimes “mutualistic goods,” sometimes “traded resources”).

**Response 8:** We have now changed “mutualistic goods” to “traded resources”.

**Reviewer #2**

Agarwal et al. studied the asymmetric fitness effects of traded resources in a multi-mutualist community. The overall study is excellent, has many strengths, and is written clearly. I only have a few comments (all well within the scope of this paper and without the need for any experiments) that could further improve the study.

**Comment 1:** I liked the overall findings. Asymmetric fitness effects led to asymmetric trait evolution. Notably, overproduction by lysine producers increased, potentially in a graded response to the expected selection pressure, while adenine overproduction showed very little change.
How does a guild change trait evolution in an asymmetric setting? In my opinion, it is important to discuss how selection might change when moving from a single strain to a combination of strains performing the same function. This is the main idea of this paper. In fact, since you have the data, it would be worthwhile to test whether the results on average trait evolution presented here would differ if considered at the genotype-specific level. I further explain these points below.

Is there any baseline within-guild genotypic variability? Do all genotypes in a guild have the same fitness, with no measurable differences among them, or is it only the case that they all overproduce adenine and underproduce lysine in similar ways, while other differences have not been measured?

**Response 1:** We appreciate the reviewer’s insightful point regarding potential within-guild genotypic variation. Each strain contributing to a guild was initiated from a single colony, minimizing within-strain genetic variation at the start of the experiment. However, to explicitly evaluate baseline differences among genotypes within the AdeOP guild, we compared ancestral strains grouped by family. Growth rate did not differ significantly among families (F_₍3,28₎_ = 2.66, p = 0.068), and no pairwise contrasts were significant (p > 0.06). These results indicate that **baseline genotypic variability for this trait was limited** within the AdeOP guild. Therefore, the guild-level patterns reported in the main text are **not driven by pre-existing fitness differences among genotypes**, but instead reflect evolutionary responses at the functional-group level. In addition, we examined baseline variation in resource overproduction among AdeOP families. Overproduction differed significantly among families (ANOVA: F_₍3,28₎_ = 6.57, p = 0.0017), with post-hoc tests indicating that genotype of 1057-1058 exhibited slightly lower overproduction levels compared to the other families (p < 0.012). Although this effect indicates some baseline variation within the AdeOP guild, the magnitude of these differences was modest, and does not alter the overall guild-level conclusions.

We performed the same analysis for the LysOP guild. Ancestral LysOP families did **not differ in growth rate** (ANOVA: F_₍3,26₎_ = 0.11, p = 0.95), and **no significant differences were detected in overproduction** (ANOVA: F_₍3,26₎_ = 2.07, p = 0.13). We observed a moderate difference in resource use efficiency (ANOVA: F_₍3,26₎_ = 5.11, p = 0.0065), driven by slightly higher values in strain 1085–1086. Together with the AdeOP analyses, these results show that **baseline variation in growth-related performance among genotypes within each guild is limited**, while modest differences are present. Therefore, the **guild-level trait evolution patterns we report are not the result of strong pre-existing fitness asymmetries among genotypes** but instead reflect evolutionary responses occurring at the functional-group level.

**Comment 2:** Is there a persistent genotype? I would not expect any particular genotype to consistently persist across communities. This can be easily tested.

**Response 2:** We assume that ‘persistent genotype” means “persistent strain”. We examined persistence at the strain level using the strain-specific data now presented in Fig. 3. While there were differences in the likelihood of persistence among strains within each guild, no strains consistently persisted across all communities. Thus, although persistence varied among strains, these differences did not drive the evolutionary patterns we report, and we do not find evidence for a single “persistent strain” or strain-specific evolutionary advantage. It is possible that genotypes within a strain could evolve and be persistent but testing that was beyond the scope of our study.

**Comment 3:** Do within-guild cheaters evolve? Is overproduction costly? If it is, could some strains within a guild reduce overproduction and act as cheaters, forcing other strains of the same guild to compensate by producing more? Again, this should be straightforward to test by comparing overproduction in specific genotypes, as opposed to comparing only averages from genotype-mixed populations before and after evolution.

**Response 3:** We examined overproduction at the level of individual genotypes within each guild to test whether any strain reduced its contribution disproportionately, which would indicate the emergence of within-guild cheaters. The strain-wise overproduction measurements (now shown in Fig. S1) revealed consistent evolutionary shifts across all genotypes within each guild. AdeOP strains showed a similar reduction in overproduction relative to their ancestral values, and LysOP strains showed a similar increase, with no strain deviating strongly from the guild-wide pattern. Therefore, we do not find evidence that any strain evolved a reduced contribution while relying on others to compensate. The observed changes instead reflect a coordinated, guild-wide evolutionary response rather than the evolution of within-guild cheaters (line 411-418).

**Comment 4:** Expectation from pairwise combination. Since some results seem to diverge from your previous pairwise study (only one genotype per guild; Wang et al. [39]), could you compare these results against expectations from the pairwise system beyond what is presented in the Discussion?
Ideally, if there is no within-guild variability, then, on average, the species in a multi-mutualist community would feel similar competition and cooperation, and thus their average persistence would match the expectation from the pairwise system. You can easily test this by comparing your pairwise system and multi-mutualist community results side by side. If this comparison is not readily feasible, I understand.

**Response 4:** We agree that comparing to expectations from our previous pairwise system is informative. However, a direct numerical comparison is not appropriate because (i) the pairwise study used a single genotype per guild while our communities contain four per guild, introducing within-guild competition and frequency-dependent effects; (ii) our outcome is persistence across replicate communities (binomial), not continuous growth/efficiency, so the mapping from pairwise dynamics to multi-member persistence is non-linear (iii) the studies were done at different times and with different media, researchers, and other factors that could influence the results. The reviewer’s question is a great one and as part of another study we are going to directly compare the pairwise and 4x4 communities since we are evolving them at the same time.

**Comment 5:** In addition, because of likely stochastic extinction in each transfer window, the selective pressure on a strain with low abundance would be to focus more on survival and could contribute to the reduced resource-use efficiency in AdeOP (a 13% decrease in obligate and 3% in supplemented), while the same did not happen to LysOP (a 5% decrease in obligate and 3% in supplemented). This can also be tested from the available data.
Modeling opportunity for the future. This experimentally grounded system, together with the evolution-specific results from this study, provides an ideal foundation for a computational model that can quantitatively dissect the remaining ecological dynamics and illuminate complex interaction rules beyond net outcomes.

**Response 5:** We thank the reviewer for this suggestion. We agree that repeated population bottlenecks may have imposed stronger selection on AdeOP strains to prioritize survival under resource limitation, potentially contributing to the greater reduction in resource-use efficiency observed in AdeOP relative to LysOP strains. We have now added this interpretation to the Discussion, and added a note that this system provides opportunities for future modeling to examine the role of extinction risk and survival-based selection in shaping mutualist evolution.

Line 414-418: Because AdeOP strains often occurred at lower abundances and were more prone to stochastic extinction during serial transfers, selection may have favored survival-oriented traits over efficient resource production. In this context, maintaining viability under severe resource limitation may have had greater fitness benefits than maximizing the mutualistic exchange.

Line 464-466: This system also provides a strong foundation for future modeling work aimed at quantifying how extinction risk and survival-based selection pressures shape evolutionary trajectories in multi-partner mutualisms.

**Minor suggestions:**

**Comment 6:** Line 308: Increased by 10%??

**Response 6:** We agree with the reviewer. We have now made the corrections in line 327-329:

**“**On average, overproduction by adenine mutualists reduced by 1 % in the obligate environment, while it increased by 10% in the environment with external resources.”

**Comment 7:** On average, lysine overproduction increased by 4% when external adenine and lysine were present in the media and by 16% in obligate media. Strains evolved in media without external resources overproduced lysine more than the ancestral strains, but less than the strains which evolved in media with external resources present (F2,88= 4.66, p= 0.012).
It seems inconsistent to me. Should it not instead be, “Strains evolved in media with external resources overproduced lysine more than the ancestral strains, but less than the strains that evolved in media without external resources present”?

**Response 7:** We agree with the comment. We have now corrected the sentence as per suggestion (line 331-334)

**Comment 8:** In Fig. 3, AdeOP1 represents the arbitrarily assigned first member of the guild, right? If so, the figure must either come from a specific community or represent an average across combinations. Why does AdeOP1 appear significantly different from the others? Why are there no error bars if this represents an average?

**Response 8:** Thank you for pointing this out. AdeOP1 is not an arbitrary member but refers to a specific adenine-overproducing strain included in the experiment. To avoid confusion, we now clarify this in the figure caption and Methods. The values shown in Fig. 3 represent the mean persistence of each strain across replicate communities, and we have now added error bars representing the binomial standard error of the proportion (calculated as √(p(1−p)/n), where p is the fraction of communities in which the strain persisted and n is the total number tested). Adding these error bars clarifies that the observed differences among AdeOP or LysOP strains reflect variation across replicates.

**Comment 9:** In Fig. 4, could you make the x-axis range the same in both?

**Response 9:** We appreciate the reviewer’s suggestion. However, the two strains show markedly different survival dynamics under starvation. AdeOPs reach extinction by approximately 72 hours, and therefore no further data could be generated beyond this time point. In contrast, LysOPs maintain viability for a longer duration, allowing us to extend the assay. Because the total experimental time span is biologically constrained by the survival limits of each strain, it is not feasible to use an identical x-axis range for both. Presenting each strain over its actual survival timeframe provides the most accurate representation of their physiological differences.

**Comment 10:** Figures 5, 6, and 7 could be combined into a single figure. This would help readers view all trait-evolution measurements in one place. Units are missing on all three y-axes. It would be helpful to use the same y-axis range, at least in the growth-rate figure.

**Response 10:** We have now combined figure 5, 6 and 7 into a single figure. The y-axes in all three figures are basically OD at 600nm. I have mentioned that now in the axes.

**Comment 11:** Optional: Could you add a panel in Fig. 1 that visually demonstrates the asymmetric effects of adenine and lysine starvation on their dynamics compared to the wild type, coupled with the corresponding adenine and lysine concentrations in the medium? This would make the conceptual asymmetry more intuitive.

**Response 11:** We understand reviewer’s suggestion. After careful consideration, we decided not to add a new figure panel because introducing a cartoon of resource dynamics alongside the existing experimental workflow would substantially increase visual complexity and potentially obscure the central purpose of Fig. 1. Instead, we have clarified the conceptual asymmetry directly in the main text (Lines 116-137) and expanded the explanation of how adenine and lysine starvation differentially influence cellular physiology, survival, and resource-release dynamics. We believe these additions communicate the underlying asymmetry more clearly without overburdening the figure layout. We hope the reviewer finds these revisions satisfactory.

**Comment 12:** Also, please ensure that all figures appear at high resolution in the published version.

**Response 12:** We will ensure that all figures are prepared and submitted in high resolution, suitable for publication quality.

**Comment 13:** Overall, this is an excellent study. I believe the revisions will make the paper more insightful and complete.

**Response 13:** We thank you for the positive feedback. We appreciate the reviewer’s encouraging remarks and are pleased that the revisions have improved the clarity and depth of the manuscript.

**Reviewer #3**

**Comment 1:** In methods section, a bit more discussion on the genetic backgrounds of the strains will be helpful.

**Response 1:** We have now added the details on genetic background in the method section (Line 101-113).

**Comment 2:** Effective population size during the evolution experiment should be mentioned so that the readers can have an idea about the strength of selection.

**Response 2:** We appreciate the reviewer’s suggestion. We have now clarified the population dynamics during the evolution experiment to provide a better sense of the strength of selection. Communities were propagated by serial transfer every 48 hours, and each transfer involved moving 10% of the culture volume into fresh medium. Cultures consistently reached high cell densities (OD_600_ ≈ 8) prior to transfer. Because the transfer volume was relatively large and populations expanded substantially between passages, the communities did not experience strong population bottlenecks. Consequently, the effective population size remained sufficiently large throughout the experiment for selection to act reliably on heritable variation. This information has been added to the Methods section (line 170-178). The transfer regime, however, did serve to reduce the number of cells per unit volume and did reduce the amount of mutualistic goods right after a transfer event.

**Comment 3:** Why single colonies of individual strains were used for phenotyping after evolution experiment? This would remove genetic and phenotypic diversity. Only the fixed mutations will be seen, and their phenotypes will be measured.

**Response 3:** We understand the reviewer’s concern. A single colony was used only to initiate each evolution line. However, for all post-evolution phenotyping assays, we did not use a single isolate. Instead, we pooled five independent colonies from each evolved population. This approach preserves both the genetic and phenotypic diversity present in the evolved lines and prevents our measurements from being limited to a single fixed genotype.

**Comment 4:** 1% lysine - v/v?

**Response 4:** We have now mentioned the details in line 230-232. “Ancestral and evolved strains of AdeOPs were diluted in SD medium containing 1% lysine (90 mg/L) and LysOPs were grown in SD medium containing 1% adenine (40 mg/L).”

**Comment 5:** Is it possible to directly measure lysine and adenine conc. in the filtrates?

**Response 5:** Yes, there is a possibility to directly quantify absolute concentrations of adenine and lysine. However, in this study our objective was to evaluate the **biologically accessible** fraction of these metabolites rather than their absolute concentrations. For this reason, we employed a growth-based bioassay, which measures how much adenine or lysine can actually be utilized by a recipient auxotrophic strain. This approach captures both metabolite release and usability, providing a physiologically meaningful estimate of resource availability under mutualistic conditions. Additionally, growth-based metabolite quantification is a standard approach in studies involving *Saccharomyces cerevisiae* auxotroph interactions (Vidal et al. 2020), allowing us to maintain methodological comparability with prior work. Our focus here is therefore on **relative differences** in accessible metabolite levels across treatments rather than on absolute concentration values.

**Comment 6:** The statements in lines 303 and 307 seem to be contradicting each other.

**Response 6:** We agree with the reviewer. We have now made the corrections in line 327-329:

**“**On average, overproduction by adenine mutualists reduced by 1 % in the obligate environment, while it increased by 10% in the environment with external resources.”

**Comment 7:** Line 317 - strains

**Response 7:** We have now changed “stains” to “strains” (line 337).

**Comment 8:** Decreased resource use efficiency in AdeOP strains - I presume that this is for lysine use. However, we see lysine overproduction in lysOP strain. Is it possible that the reduction in RUE in AdeOP is caused by lysine overproduction?

**Response 8:** The reduction in resource use efficiency (RUE) observed in the AdeOP strain cannot be driven by lysine overproduction in LysOP. It might be because of the **metabolic burden associated with adenine overproduction**. In contrast, lysine overproduction in LysOP is mediated through a pathway with a **lower energetic cost**, and therefore does not impose a comparable efficiency penalty. Thus, the asymmetry in RUE reflects **intrinsic differences in the metabolic cost of adenine versus lysine biosynthesis**, rather than lysine overproduction reducing lysine utilization efficiency in AdeOP.

**Comment 9:** It is quite puzzling to see reduction in resource use efficiency in LysOP and AdeOP communities in obligate condition. Why would this happen? It would be good if the authors can discuss this point along with some plausible explanations.

**Response 9:** We thank the reviewer for raising this thoughtful point. In the obligate condition, there are in general less mutualistic goods available, especially in the initial hours after each serial transfer. This situation is alleviated when external mutualistic goods are provided. As such, there is usually selection to grow fast rather than to maximize RUE (see Bachmann et al, 2106, Public goods and metabolic strategies). We would expect both strains to reduce RUE to maximize growth and this tradeoff is well documented in microbes.

We have added the following to the discussion (line 420-423):

Additionally, the serial propagation transfer protocol also reduced traded resources in the environment, especially for the obligate conditions, thus favoring strains that were better at growing faster rather than being more efficient [39]. This often comes at the expense of RUE [40].

**References**

39. Bachmann H, Bruggeman FJ, Molenaar D, Dos Santos FB, Teusink B. Public goods and metabolic strategies. Current opinion in microbiology. 2016 Jun 1;31:109-15.

40. Frank SA. The trade‐off between rate and yield in the design of microbial metabolism. Journal of evolutionary biology. 2010 Mar 1;23(3):609-13.

**Comment10:** Sub-headings in the results could be changed to statements that summarize the results discussed in that section

**Response 10:** We have now changed sub-headings of the results section.
