# Supplementary material for: Differences in the fitness effects of traded resources shape traits and persistence in multi-mutualist communities
Source: PLoS One. 2026 Feb 3;21(2):e0340707. doi: 10.1371/journal.pone.0340707 (PMC12867262; doi:10.1371/journal.pone.0340707)
Supplement: Attachment — Submitted filename: Response_to_Reviewers_auresp_2.docx [file pone.0340707.s007.docx]

**Comments to Reviewers**

We thank the reviewers for their careful re-evaluation of our manuscript and for accepting most of our responses from the previous revision. We appreciate the constructive feedback provided in the second round of review. Below, we address the remaining comments and have revised the manuscript accordingly. We believe that these additional clarifications and modifications have further strengthened the clarity and rigor of the study.

**Reviewer #1**

We thank reviewer for accepting our responses and changes.

**Reviewer #2**

The authors have adequately addressed all of my major and minor concerns, and I recommend the paper for publication. I congratulate the authors on a well-executed study. I have one minor change request that does not require further review.

**Comment 1:** Figure 4 (Comment 9): I understand and agree with your concern, but my suggestion was purely graphical. Please keep the same x-axis limits to aid visual comparison, even if one curve ends earlier (the remaining region can be left blank). No additional measurements required.

**Response 1:** We have revised Figure 4 in accordance with the reviewer’s suggestion by modifying the x-axis to improve visual comparison. Specifically, rather than leaving the terminal time points blank, we have set the proportion to zero, as the proportion of live colonies reaches zero after 72 hours. This provides a clearer and more accurate representation of the observed dynamics across time.

**Comment 2:** Minor check: Please verify the reported F values around lines 301–303.

**Response 2:** We thank the reviewer for the comment. After verifying, we have made changes to the lines (now line 295-298 in file with track changes on):

“A two-way ANOVA revealed a significant main effect of media type (F₁,₅₄ = 42.54, p < 0.001). In contrast, the main effect of overproducer type was not significant (F₁,₁₆ = 0.00, p = 1.00). Importantly, a significant interaction between media type and overproducer type was detected (F₁,₅₄ = 15.78, p < 0.001), indicating that the effect of media on persistence depends on the overproduction classification (mutualist type).”

**Reviewer #3**

**Comment 1:** The authors have addressed all my previous comments satisfactorily.
However, in the caption of figure 5, the authors should mention the name of the statistical test that is being performed.

**Response 1:** We have now mentioned the name of statistical test that was performed to make figure 5. We have added the following line in the caption of figure 5 (Line 633-634 in file with track changes on):

“Statistical analyses were performed using linear mixed-effects models, with significance of fixed effects assessed using F tests (lmerTest package in R).”
